# Exploring ncRNAs in Alzheimer’s disease by miRMaster

**DOI:** 10.18632/oncotarget.14054

**Published:** 2016-12-20

**Authors:** Tobias Fehlmann, Eckart Meese, Andreas Keller

**Affiliations:** Department of Clinical Bioinformatics, Saarland University, Saarbrücken, Germany

**Keywords:** miRNA, microRNA, nc-RNA, NGS, sequencing, Neuroscience

Circulating biomarkers for the diagnosis of Alzheimer’s Disease (AD) such as amyloid-β levels are already applied in clinical care. New markers, especially marker panels that can be measured from blood and specifically focusing at early diagnosis, are under development [1]. Previously, we published miRNA signatures in Alzheimer’s Disease that have been discovered by high-throughput sequencing (also denoted as next-generation sequencing, NGS) [2, 3]. These studies focused on the quantification of known human miRNAs. Sequencing the fraction of all small RNAs in a specimen, however, allows not only for investigating canonical miRNAs but also enables prediction of new miRNAs, isoforms of miRNAs and even provides first insights in other RNA types.

We developed a tool for the comprehensive analysis of small non-coding RNAs, primary miRNAs, starting from NGS raw data. Our web-based program miRMaster is free for academic users: www.ccb.uni-saarland.de/ mirmaster. Beyond the core functionality of analyzing known miRNAs, predicting isomiRs, discovery of mutations in miRNAs and identifying new miRNAs, we implemented modules for quantification and comparison of other nucleic acid resources. All sequencing reads provided to miRMaster are mapped to the NCBI RefSeq bacteria and viruses collection, Ensembl ncRNAs, piRBase and GtRNAdb. Altogether, the small RNA reads are mapped against 66,521 references comprising 15.76 GB sequences.

**Figure 1:**
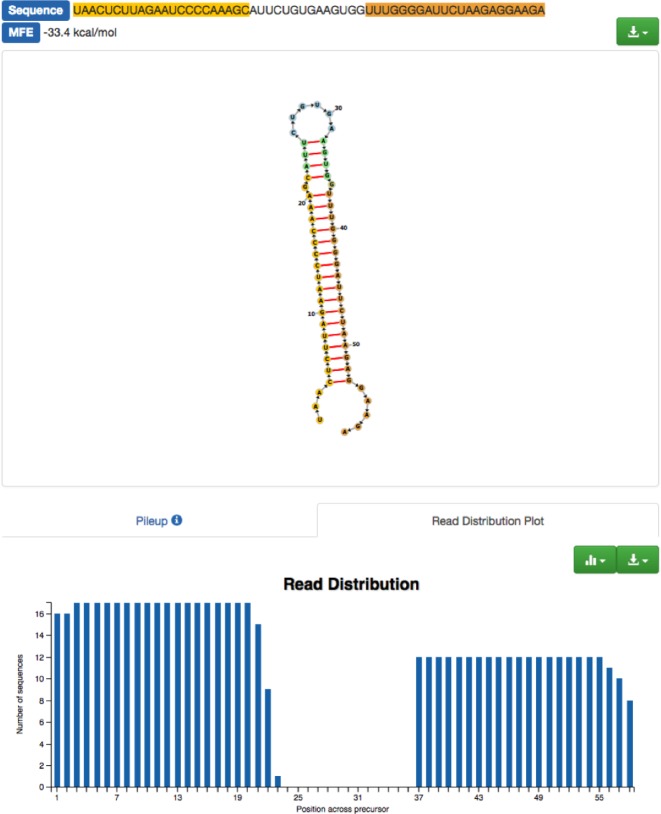
example of a novel miRNA candidate in miRMaster

The pre-processing, upload and complete online analysis of 70 AD samples and controls comprising 1.2 Billion short sequencing reads has been performed in one hour, highlighting the speed of miRMaster. Of all reads, 1.13 Bn mapped to the human genome and 1.07 Bn to known human miRNAs, leaving 60 million reads mapping to the human genome but not to annotated miRNAs. These reads contain potentially novel miRNAs or other RNA types. With respect to novel candidates, one example is presented in Figure 1. The graphic shows the conformation of a predicted precursor and the reads mapping to this precursor in one sample. Among the significant results in comparing AD versus controls following adjustment for multiple testing that are no miRNAs we found 11 tRNAs. Top scoring was tRNA-Val-AAC-5-1 with p-value of 0.025 and 3.1 RPM in Alzheimer cases versus 1.3 RPM in controls. Additionally, we observed one significant piRNA: piR-hsa-28876 (p-value of 0.015). Also one snoRNA, SNORD88A remained significant following adjustment for multiple testing (p-value of 0.023) and was up-regulated more than two-fold in AD cases. The same holds true for snRNAs with RNU4-46P being significant (*p*-value of 0.014). For all other resources (lincRNAs, rRNAs, scaRNAs and the bacterial and viral genome collection from NCBI) we only discovered hits that were significant prior to adjustment for multiple testing.

Of course, the presented results for matches of small RNA sequencing reads to non-miRNA nucleic acid resources can only serve as a starting point for further analyses. These matches can subsequently be related to the candidate hits such as significant tRNAs. The same holds true for the prediction of novel miRNAs, which also have to be tested for their functionality. This is especially important since different sequencing techniques relying on different basic principles may lead to different results, as for example found by a comparison between Illumina HiSeq and BGISEQ-500 [4]. Since the flood of potentially new miRNAs cannot be verified using low-throughput approaches, prioritizing and pre-selection is urgently required in order to reduce the number of false positives [5]. Overcoming respective technical challenges in small RNA analysis using high-throughput sequencing and validation of candidates are important steps in the demanding translation of miRNA signatures to clinical care [6].
